# AdeABC Efflux Pump Controlled by AdeRS Two Component System Conferring Resistance to Tigecycline, Omadacycline and Eravacycline in Clinical Carbapenem Resistant *Acinetobacter nosocomialis*

**DOI:** 10.3389/fmicb.2020.584789

**Published:** 2020-11-02

**Authors:** Yi-Tzu Lee, Hsing-Yu Chen, Ya-Sung Yang, Yu-Ching Chou, Tein-Yao Chang, Wei-Jane Hsu, I-Chieh Lin, ACTION Study Group, Jun-Ren Sun

**Affiliations:** Yea-Yuan Chang (National Yang-Ming University Hospital, Yilan, Taiwan), Y-SY (Division of Infectious Diseases and Tropical Medicine, Department of Internal Medicine, Tri-Service General Hospital, National Defense Medical Centre, Taipei, Taiwan), Chung-Ting Chen (Department of Emergency Medicine, Taipei Veterans General Hospital, Taipei, Taiwan), Yuag-Meng Liu (Changhua Christian Hospital, Changhua, Taiwan), Shu-Chen Kuo (National Institute of Infectious Diseases and Vaccinology, National Health Research Institute, Maoli County, Taiwan), Chang-Pan Liu (MacKay Memorial Hospital, Taipei, Taiwan), Te-Li Chen (Graduate Institute of Life Sciences, National Defense Medical Centre, Taipei, Taiwan), and Y-TL (Taipei Veterans General Hospital, Taipei, Taiwan); ^1^Department of Emergency Medicine, Taipei Veterans General Hospital, Taipei, Taiwan; ^2^Faculty of Medicine, School of Medicine, National Yang-Ming University, Taipei, Taiwan; ^3^Department of Medical Techniques, Taipei City Hospital Ren-Ai Branch, Taipei, Taiwan; ^4^Division of Infectious Diseases and Tropical Medicine, Department of Internal Medicine, Tri-Service General Hospital, National Defense Medical Center, Taipei, Taiwan; ^5^School of Public Health, National Defense Medical Center, Taipei, Taiwan; ^6^Institute of Preventive Medicine, National Defense Medical Center, Taipei, Taiwan

**Keywords:** tigecycline, *Acinetobacter*, omadacycline, eravacycline, efflux pump

## Abstract

Carbapenem-resistant *Acinetobacter nosocomialis* (CRAn) is a significant public health concern. Tigecycline non-susceptible CRAn (Tn-CRAn) isolates have emerged worldwide. Tigecycline resistance is mainly related to the overexpression of AdeABC efflux pump controlled by AdeRS two-component system (TCS). Two novel tetracycline derivatives, omadacycline and eravacycline, may present a treatment option for CRAn. This study investigated the *in vitro* antimicrobial activity of tigecycline, omadacycline and eravacycline against clinical CRAn isolates and the contribution of efflux pumps in their resistance. Eighty-nine clinical CRAn isolates, including 57 Tn-CRAn isolates were evaluated for minimum inhibitory concentrations (MICs) by the broth microdilution. The relationship between the antimicrobial resistance and efflux pump expression was assessed by their responses to the efflux pump inhibitor 1-(1-naphthylmethyl)-piperazine (NMP). The contribution of the AdeABC efflux pump in their resistance was determined by the complementation of the AdeRS two-component system in wild-type, *adeRS* operon and *adeB* gene knockout strains. Among the 89 isolates, omadacycline and eravacycline MICs were correlated closely with those of tigecycline. They demonstrated improved potency, based on MIC_90_ values, by showing a 4 to 8-fold greater potency than tigecycline. The synergetic effects of tigecycline, omadacycline and eravacycline with NMP were observed in 57 (100%), 13 (22.8%), and 51 (89.5%) of Tn-CRAn isolates, respectively. Further analysis showed that the laboratory strain carrying the Type 1 *adeRS* operon increased the tigecycline, omadacycline and eravacycline MICs by 4–8-folds, respectively. Eravacycline demonstrated improved potency over tigecycline against populations of CRAn, including Tn-CRAn isolates. The over-expression of AdeABC efflux pumps was directly activated by the AdeRS two-component system and simultaneously reduced the susceptibilities of tigecycline, eravacycline, and omadacycline. Omadacycline and eravacycline MICs were correlated closely with those of eravacycline.

## Introduction

*Acinetobacter* species has become a major nosocomial pathogen associated with high mortality in immunocompromised patients on account of its rapid acquisition of resistance (Ayoub Moubareck and Hammoudi Halat, 2020). When *Acinetobacter* spp. develops extended drug resistance to sulbactam, tigecycline, and colistin, the antimicrobial choices become scarce and difficult (Montana et al., 2015). New and novel antimicrobial agents are therefore needed.

*Acinetobacter nosocomialis* is an emerging opportunistic pathogen that is usually grouped into the *Acinetobacter calcoaceticus-baumannii* complex (Acb complex) (Vijayakumar et al., 2019). In Asia, the carbapenem resistant rate among infections caused by *A. nosocomialis* is as high as 30% (Chen et al., 2018; Singkham-In and Chatsuwan, 2018; Chen et al., 2019). Tigecycline has been regarded as one of the final armamentaria against carbapenem resistant *A. nosocomialis* (CRAn). Unfortunately, tigecycline non-susceptible *A. nosocomialis* has also increasingly emerged in recent years (Yang et al., 2019).

Resistance-nodulation-cell division (RND) efflux pumps are ubiquitous in gram-negative bacteria and have been shown to play an important role in antimicrobial resistance (Coyne et al., 2011; Li et al., 2015b). It has been shown that three efflux pumps, AdeABC, AdeFGH, and AdeIJK, are associated with tigecycline resistance in *Acinetobacter* species (Coyne et al., 2011). The expression of the AdeABC is controlled by the AdeRS two-component system (TCS). The expression of AdeFGH and AdeIJK is controlled by AdeL, a LysR-type transcriptional regulator and AdeN, a TetR-like transcriptional regulator, respectively (Xu et al., 2019). Our previous study has demonstrated that the overexpression of the AdeABC efflux pump plays a major role in exporting tigecycline in tigecycline non-susceptible *A. nosocomialis* (Yang et al., 2019). Type 1 AdeRS TCS was found to be associated with tigecycline non-susceptible in clinical *Acinetobacter nosocomialis* in isolates (Yang et al., 2019). However, the correlation between the Type 1 AdeRS TCS pattern and tigecycline resistance is still uncertain and requires further investigation.

Omadacycline and eravacycline are novel tetracycline derivatives similar to tigecycline, and they were found to have broad spectrum activity against various multi-drug resistant pathogens (Zhanel et al., 2018; Karlowsky et al., 2019; Kaushik et al., 2019). Because of the structural resemblance to tigecycline, omadacycline and eravacycline are believed to have good activity against clinical CRAn. In addition, there were no reports indicating omadacycline and eravacycline susceptibilities among tigecycline non-susceptible CRAn (Tn-CRAn). The aim of this study was to examine the antimicrobial susceptibilities of omadacycline and eravacycline against the emerging CRAn, in comparison with tigecycline. Furthermore, we also investigated the role of efflux pump-mediated resistance to omadacycline and eravacycline.

## Materials and Methods

### Bacterial Strains

The bacterial strains and plasmids used in this study are listed in Table 1. Between 2012 and 2018, clinical CRAn isolates were collected from AntimiCrobial studies in Taiwan Operating Network (ACTION), which included six medical centers located in different parts of Taiwan, including (alphabetically) the Changhua Christian Hospital (CCH) in Central Taiwan, Kaohsiung Medical University Hospital (KMUH) in southern Taiwan, Mackay Memorial Hospital (MMH) in Northern Taiwan, National Taiwan University Hospital (NTUH) in Northern Taiwan, Taipei Veterans General Hospital (TVGH) in Northern Taiwan, Tri-Service General Hospital (TSGH) of the National Defense Medical Center (NDMC) in Northern Taiwan and National Institute of Infectious Diseases and Vaccinology, National Health Research Institute (NHRI) in Northern Taiwan. All isolates were identified by MALDI-TOF (matrix-assisted laser desorption ionization-time of flight) and confirmed by a sequence analysis of the *rpoB* gene (Bartual et al., 2005). The isolates were subjected to multi-locus sequence typing (MLST) according to the Pasteur scheme (Diancourt et al., 2010). The genetic relationship among the MLST types of Tn-CRAn isolates was reconstructed using the Phyloviz program.^1^

**TABLE 1:** Bacterial strains and plasmids used in this study.

Strains or plasmids	Relevant characteristics
***Acinetobacter nosocomialis* strains**	
An wt	*A. nosocomialis* reference strain ATCC17903
AnΔadeRS	Derived from ATCC17903. *adeRS* operon deletion
AnΔadeB	Derived from ATCC17903. *adeB* gene deletion
**Plasmids**	
pS01	*E. coli*-*Acinetobacter* shuttle plasmid; mini-CTX::lacIq-PT7-lacZ
pRS-AnadeRS (wt)	Derived from pRS, mini-CTX:: adeRS-Pade-lacZ (adeRS from ATCC 17903)
pRS-AnadeRS (Type1)	Derived from pRS, mini-CTX:: adeRS-Pade-lacZ (adeRS from clinical Tn-CRAn isolate)

*MIC, Minimum inhibitory concentration.*

### Antimicrobial Susceptibility and Efflux Pump Inhibitor Tests

Antibiotic susceptibility testing was performed using Sensititre GNX2F (Thermo Fisher Scientific, Waltham, MA, United States) following the manufacturer’s protocols. Minimum inhibitory concentrations (MICs) of tigecycline, omadacycline and eravacycline were determined by broth microdilution. The U.S. Food and Drug Administration (FDA) recommendation for tigecycline susceptibility breakpoints of *Enterobacteriaceae* (susceptible MIC≤2 mg/L; intermediate >2 and <8 mg/L; resistant ≥8 mg/L) was used as the MIC interpretation criteria (Yang et al., 2019). Omadacycline and eravacycline were currently without breakpoints. *Escherichia coli* ATCC 25922 was utilized as a quality control for each series of isolates tested. The effect of 1-(1-naphthylmethyl)-piperazine (NMP) on antibiotic activity was determined by the Mueller-Hinton broth (MHB) in the presence and absence of NMP (50 mg/L), respectively. The NMP effects were interpreted and reported as follows: good effect for a 4-fold or more reduction and poor effect for a less than 2-fold reduction in the MIC value (Yang et al., 2019).

### Construction of Deletion Mutations and *adeRS* Transforms

ATCC17903ΔadeRS (AnΔadeRS) and ATCC17903ΔadeB (AnΔadeB) were unmarked deletion mutants created by a previously described method for generating markerless deletions in *A. nosocomialis* strain ATCC17903 (An wt) as suggested in a previous study (Sun et al., 2017). Primers are shown in Table 2. Suicide plasmid pMo130-TelR was used for deleting the *adeRS* and *adeB* operons. Approximately 1-kb fragments upstream and downstream from the target genes were amplified and cloned in pMo130-TelR. Successful ATCC17903 transformants were first selected on LB agar plates containing 10 mg/L kanamycin (first crossovers). Kanamycin-resistant clones were isolated, and single homologous recombination events were screened for by PCR. Kanamycin -resistant clones were then plated on an LB agar containing 10% sucrose to be selected for the deletion of the *adeRS* operon or *adeB* gene by a second crossover and allelic replacement.

**TABLE 2:** Oligonucleotides used in this study.

Primer name	Sequence	Features/purpose
An_adeRS_r_NaeI	AAGAGCCGGCAGTGAAGAGTTGTAACGCTAAGG	Cloning *adeRS* operon and promoter with engineered NaeI and BamHI site into pRS to form pRS- adeRS.
An_Apromoter_f_BamHI	CCGAGGATCCAAAAGATGCTTTTGCATACTGTC	
pMo130Tel F	TTTACCACGACCGCATTCTC	Primers specific to pMo130TelR DNA flanking the Up and Down insert; PCR product not present after single and double crossover.
pMo130Tel R	AAATAGGCGTATCACGAGGC	
AdeAUp_f (Pst1)	ACTCTGCAGGCTGATGTCGCTCAAATGAAAGCA	PCR of UP fragment containing partial AdeB region.
AdeAUp_r (BamH1)	ATGGATCCCGGAGCTACACTTGGAAAGC	
AdeBDwn_fover (BamH1)	GCTTTCCAAGTGTAGCTCCGGGATCCAT	PCR of DOWN fragment containing partial AdeB region.
	TGACCGTGAAAAGCTGAGTGCACTT	
AdeBDwn_r (Sph1)	CAAGCATGCGCCAGAATGGTTGCTGAAATCAT	
AdeRUp_f (Pst1)	ACTCTGCAGTTCATTTGAGCGACATCAGC	PCR of UP fragment containing partial AdeR region.
AdeRUp_r (BamH1)	GATGGATCCGCCTGAACTCTAGCGACCAC	
AdeSDwn_fover (BamH1)	GTCGCTAGAGTTCAGGCGGATCCATCTTGCGAAGCGTTTCATT	PCR of DOWN fragment containing partial AdeS region.
AdeSDwn_r (Sph1)	CAAGCATGCCCTAAACCTGTACCGCCAAA	

The plasmid pS01 was digested with *Sma*I and *BamH*I to delete its lacIq gene and T7 early gene promoters. The digested plasmids were ligated with the respective gene fragments to generate a series of recombinant pRS clones, including pRS-AnadeRS (wt) and pRS-AnadeRS (Type1). Recombinant plasmids were introduced by electroporation into AnΔadeRS and AnΔadeB and selected on LB agar plates containing 5 mg/L tetracycline.

### Quantitative Real-Time PCR

The transcription levels of *adeB*, *adeJ*, and *adeG* genes were measured by quantitative real-time PCR assays as suggested in a previous study (Yang et al., 2019). ATCC 17903 transforms were grown in Luria-Bertani (LB) broth until mid-log phase. Total RNA was extracted using an RNeasy Mini Kit (Qiagen Sciences, Germantown, MD, United States). Reverse transcription was performed using a QuantiNova Reverse Transcription Kit (Qiagen). Real-time PCR was performed with the QuantiNova SYBR Green PCR Kit (Qiagen). The mRNA of *rpoB* gene was used as a control and ATCC 17903 as a reference to the standard expression level.

### Statistical Analyses

Statistical analysis was carried out using the SPSS version 20.0 software package. The calculation of statistical differences between various groups was based on the Fisher’s exact test. Pairs of outcomes (Correlation of MICs between two antibiotics) were examined for evidence of monotonic associations using Spearman’s correlation coefficients. Differences were considered statistically significant when *p* < 0.05.

## Results

### Susceptibilities Among CRAn Isolates

**FIGURE 1:**
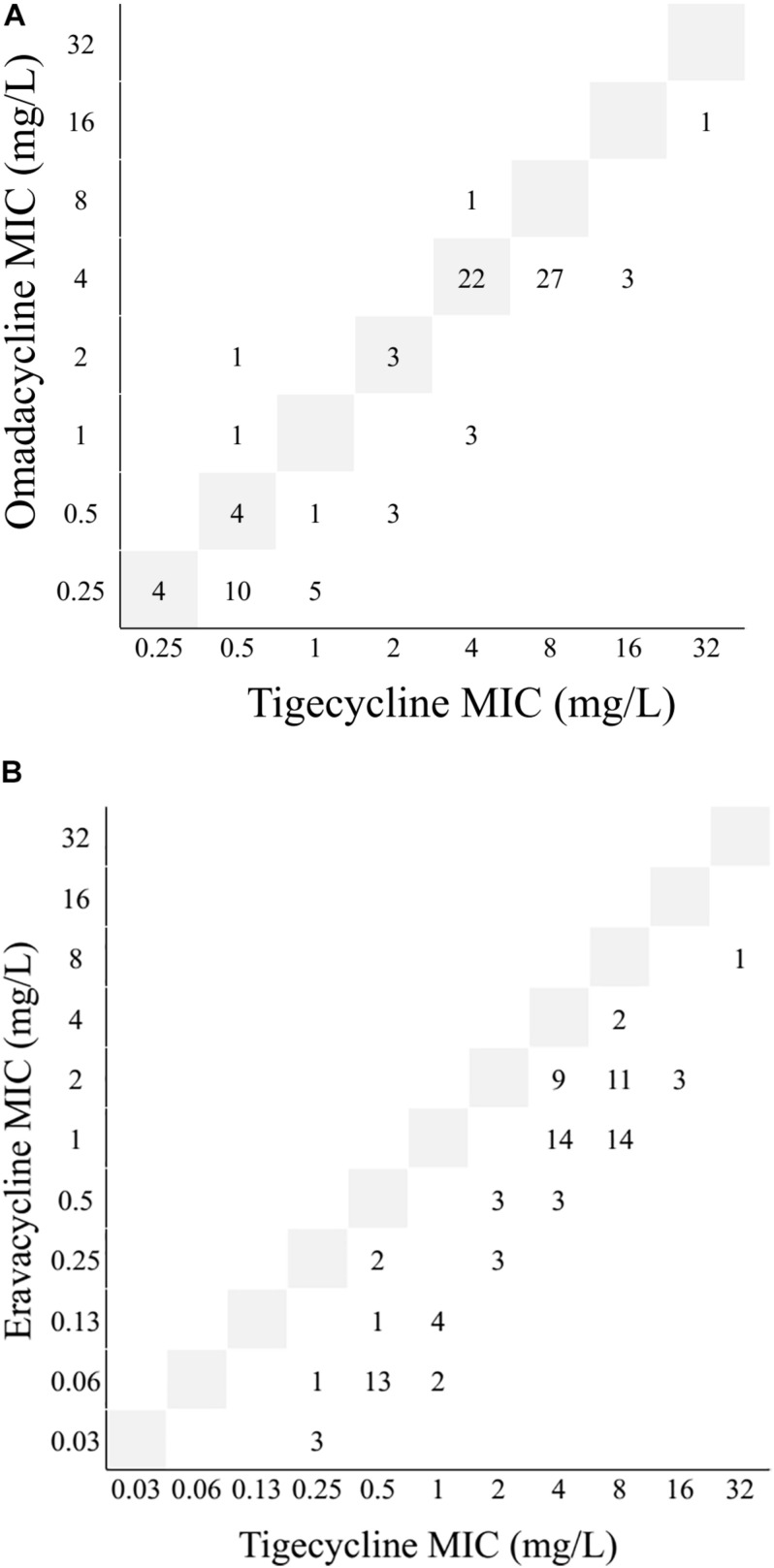
Interrelationship between minimum inhibitory concentrations (MICs) of omadacycline, eravacycline and tigecycline against 89 *Acinetobacter nosocomialis* isolates. **(A)** omadacycline vs. tigecycline. **(B)** eravacycline vs. tigecycline. Gray boxes represented the line of equivalence. Numbers below this line indicated omadacycline or eravacycline was more active, and numbers above indicate tigecycline was more active.

A total of 89 clinical CRAn isolates were collected, including 57 Tn-CRAn from ACTION medical centers. The antimicrobial susceptibilities of Tn-CRAn isolates are shown in Supplementary Table S1. Multilocus sequence typing revealed the Tn-CRAn isolates belonged to three sequence types (ST): ST410 (55 isolates), ST68 (1 isolate) and ST1272 (1 isolate) (Supplementary Figure S1). Tigecycline susceptible CRAn (Ts-CRAn) isolates were randomly selected with the following STs: ST1272 (14 isolates), ST433 (6 isolates), ST410 (6 isolates), ST68 (4 isolates) and ST217 (2 isolates). The overall tigecycline MICs of CRAn isolates ranged from 0.25 to 32 mg/L; omadacycline, 0.25 to 16 mg/L; and eravacycline, 0.03 to 8 mg/L (Table 3). Among the 57 isolates, 34 displayed lower omadacycline MICs than tigecycline MICs (Figure 1A) and all displayed lower eravacycline MICs than tigecycline MICs (Figure 1B). Correlation analysis was carried out to test the correlation between levels of resistance to the three tetracycline derivatives (Table 4). There was a strong correlation among MICs for the three antibiotics (Spearman’s correlation coefficients = 0.829 to 0.852, both *P* < 0.05). Taken together, these data indicate that omadacycline and eravacycline showed a positive correlation with tigecycline activity, but they were not associated with the MLST profile. Eravacycline demonstrated improved potency over tigecycline, based on MIC_90_ values, showing a 4-fold and 4- to 8-fold greater potency against populations of Tn-CRAn and Ts-CRAn, respectively (Table 5).

**TABLE 3:** Minimum inhibitory concentration distributions of tigecycline, omadacycline, and eravacycline in relation to multi-locus sequence typing types.

	No. of isolates with MLST pattern at various MICs (mg/L)										
Antibiotics and MLST pattern (n)	0.03	0.06	0.13	0.25	0.5	1	2	4	8	16	32
**Tigecycline**											
ST410 (61)					4		2	25	26	3	1
ST1272 (15)				1	10	2	1	1			
Other type (13)^a^				3	2	4	3		1		
**Omadacycline**											
ST410 (61)				1	2	4	2	50	1	1	
ST1272 (15)				11	2		1	1			
Other type (13)^a^				7	4		1	1			
**Eravacycline**											
ST410 (61)		4			5	27	22	2	1		
ST1272 (15)	1	8	2	3		1					
Other type (13)^a^	2	4	3	2	1		1				

*^*a*^ST433 (6) ST68 (5) and ST217 (2).MIC, Minimum inhibitory concentration; MLST, multi-locus sequence typing; OT, other type.*

**TABLE 4:** Correlations between minimum inhibitory concentrations of tigecycline, omadacycline, and eravacycline against carbapenem resistant *Acinetobacter nosocomialis*^a^.

	Spearman’s correlation coefficient (*P*-value)		
Antibiotics	Tigecycline	Omadacycline	Eravacycline
Tigecycline	1.000		
Omadacycline	0.852 (*P* < 0.001)	1.000	
Eravacycline	0.852 (*P* < 0.001)	0.829 (*P* < 0.001)	1.000

*^*a*^Minimum inhibitory concentrations were measured from all 89 carbapenem resistant *A. nosocomialis* isolates.*

**TABLE 5:** Minimum inhibitory concentration reduction effects of 1-(1-naphthylmethyl)-piperazine (NMP) in relation to multi-locus sequence typing types between tigecycline resistant/carbapenem resistant and tigecycline susceptible/carbapenem resistant *Acinetobacter nosocomialis*.

	Tigecycline				Omadacycline				Eravacycline			
MLST profiles	Good (≥4-folds)	Poor (≤2-folds)	MIC Range	MIC 50/90	Good (≥4-folds)	Poor (≤2-folds)	MIC Range	MIC 50/90	Good (≥4-folds)	Poor (≤2-folds)	MIC Range	MIC 50/90
**Tn-CRAn (57)**												
ST410 (55)	55		4–32	8/8	12	43	1–16	4/4	49	6	0.5–8	1/2
ST1272 (1)	1		4	4/4	1		4	4/4	1		1	1/1
OT (1)	1		8	8/8		1	4	4/4	1		2	2/2
**Ts-CRAn (32)**												
ST410 (6)	2	4	0.5–2	0.5/2		6	0.25–2	0.5/2		6	0.06–0.5	0.06/0.5
ST1272 (14)	1	13	0.2–2	0.5/1	1	13	0.25–2	0.25/0.5	1	13	0.03–0.25	0.06/0.25
OT (12)	2	10	0.25–2	1/2		12	0.25–2	0.25/0.5	2	10	0.03–0.5	0.06/0.25

*MIC, Minimum inhibitory concentration; MLST, multi-locus sequence typing; OT, other type; Tn-CRAn, tigecycline resistant/carbapenem resistant *A. nosocomialis*; Ts-CRAn, tigecycline susceptible/carbapenem resistant *A. nosocomialis.**

### Effects of NMP on Antibiotic Activity

The effects of the NMP on MICs of the three antibiotics against CRAn isolates are reported in Table 5. Among those, Tn-CRAn isolates showed better response to NMP in the three antibiotics than Ts-CRAn isolates (Fisher’s exact test; *P* < 0.05). All Tn-CRAn isolates showed a more than 4-fold decrease in eravacycline MICs with NMP. However, only seven Tn-CRAn isolates reached such reduction in omadacycline MICs with NMP. Among the NMP effects in the three antibiotics against Tn-CRAn isolates, Tigecycline and eravacycline with NMP both showed significantly more MIC reductions than omadacycline with NMP (Fisher’s exact test; *P* < 0.05).

### The Contribution of AdeRS TCS in AdeABC Efflux Pump-Mediated Resistance

To confirm the roles of AdeABC efflux pumps in the resistance to the three antibiotics, *AdeRS* operon and *adeB* gene deletion mutants were generated and tested. The sequence alignment of AdeR and AdeS sequences from Type 1 AdeRS TCS (GenBank accession number MH321430) and ATCC 17903 AdeRS TCS (wt) were shown in Supplementary Figure S2. The two amino acid differences were found between the AdeRS patterns, which contained S16N in AdeS and T137S in AdeR. The AnΔadeRS transformed with pRS-AnadeRS (Type1) showed higher MICs of the three antibiotics in comparison with pRS-adeRS (wt) [tigecycline (1 vs. 0.13 mg/L); omadacycline (2 vs. 0.25 mg/L); eravacycline (0.25 vs. 0.06 mg/L)] (Table 6). The transcription levels of *adeB* genes in AnΔadeRS with pRS-AnadeRS (Type1) were higher than that with pRS-adeRS (wt) (27.4-folds vs. 1.4-folds). The increase of omadacycline and eravacycline MIC was similar to tigecycline MIC and correlated with the expression of AdeABC efflux pump in the transformant. The transcription levels of *adeJ* and *adeG* in the two transforms were not significantly different. The MICs of the three antibiotics did not increase while being transformed with those recombinant plasmids into the AnΔadeB strain. These findings suggested that AdeABC efflux pumps medicated by AdeRS TCS played a role in the resistance to the three antibiotics in *A. nosocomialis*.

**TABLE 6:** Minimum inhibitory concentrations of tigecycline, omadacycline and eravacycline against various *Acinetobacter nosocomialis* transformants.

	MIC (mg/L)			RND efflux pump expression (fold (SD))		
Strains and plasmid	Tigecycline	Omadacycline	Eravacycline	*adeB*	*adeJ*	*adeG*
An wt	0.13	0.25	0.06	1	1	1
AnΔadeRS::pS01 (vector only)	0.13	0.25	0.06	1.0 (0.3)	1.1 (0.4)	0.8 (0.2)
AnΔadeRS::pRSANadeRS (Type1)	1	2	0.25	27.4 (7.7)	1.1 (0.6)	0.5 (0.1)
AnΔadeRS::pRSANadeRS (wt)	0.25	0.5	0.06	1.4 (0.4)	1.1 (0.4)	0.7 (0.3)
AnΔadeB::pS01 (vector only)	0.13	0.25	0.06	-	1.1 (0.5)	0.9 (0.3
AnΔadeB::pRSANadeRS (Type1)	0.13	0.25	0.06	-	1.5 (1.2)	0.6 (0.3)
AnΔadeB::pRSANadeRS (wt)	0.13	0.25	0.03	-	1.4 (1.0)	0.6 (0.1)

## Discussion

This is the first study elucidating the contribution of efflux pumps in the resistance to novel tetracycline derivative antibiotics, eravacycline and omadacycline in clinical carbapenem resistant *A nosocomialis* isolates. We found that there was a strong correlation among the MICs of the two antibiotics and tigecycline. NMP pump inhibitors reduced the MICs of tigecycline and eravacycline against Tn-CRAn isolates. Since the synergistic effect with NMP for omadacycline susceptibility was poor, it suggests that other mechanisms are contributing to omadacycline resistance. Further studies confirmed that AdeRS TCS played a role in the resistance to the three antibiotics in *A. nosocomialis*.

ST410 was found to be the most dominant type in clinical Tn-CRAn isolates in Taiwan, which is consistent with our previous studies (Chen et al., 2018; Yang et al., 2019). This may indicate that there is an unknown mechanism in ST410 that provides fitness advantage in a hospital setting, which could easily transform to carbapenem and/or tigecycline resistance. Further evaluation is needed to elucidate the relationship between ST410 and tigecycline resistance, such as whole-genome sequence analysis by next generation sequencing. MICs of omadacycline and eravacycline for the ST410 Tn-CRAn isolates were unimodally distributed, with clustering at 4 vs. 1 to 2 mg/L. In previous studies, the *A. baumannii* with higher MICs of tigecycline also had a strong positive correlation with higher eravacycline MICs, and the eravacycline MICs were generally 2-fold lower than that of tigecycline (Livermore et al., 2016; Seifert et al., 2018). In this study, we observed a strong correlation among the MICs of the three antibiotics against *A. nosocomialis*. The results suggested that the resistant mechanisms of *A. nosocomialis* against these three antibiotics might be similar due to the similar tetracycline-like structures and related to the AdeABC efflux pump.

1-(1-naphthylmethyl)-piperazine is a type of naphthyl derivative and is often used as an efflux pump inhibitor (EPI) in research (Sonnet et al., 2012; Yang et al., 2019). EPI has the ability to reverse antibiotic resistance, but is partly related to the ability to inhibit the AdeABC efflux pump (Pannek et al., 2006). In the current study, we found that omadacycline with NMP has weaker synergistic activity compared to the other two antibiotics. Moreover, we found that this was not related to the overexpression of the AdeABC efflux pump system. However, other findings about NMP under sub-inhibitory concentrations have also been reported in recent years, including reduction of the mass of preformed biofilm, inhibition of virulence factors and membrane destabilization (Bina et al., 2009; Anes et al., 2018; Anes et al., 2019). During survival in an environmental stress containing NMP, *Klebsiella pneumoniae* would initiate many genes involved in repairing and maintaining membrane homeostasis, up-regulation of other efflux systems and lipopolysaccharide modification genes (Anes et al., 2019). These findings may also suggest that sub-inhibitory concentrations of NMP not only inhibit the AdeABC efflux pump, but also drive other specific mechanisms of resistance to omadacycline.

The resistant mechanisms against tigecycline in *Acinetobacter* spp. included RND efflux pump overexpression, modifications at the ribosomal binding site, and enzymatic inactivation by tetracycline monooxygenase TetX (Chen et al., 2014; Beabout et al., 2015; Li et al., 2015a; Wang et al., 2019; Yang et al., 2019). Although the plasmid mediated TetX gene has been found to confer resistance to tigecycline, the overexpression of chromosomal RND efflux pumps, especially the AdeABC efflux pump is still a widespread tigecycline resistance determinant (Wang et al., 2019; Yang et al., 2019). The AdeABC efflux pump has been demonstrated to be present in most clinical *A. nosocomialis* and *A. baumannii* isolates (Yang et al., 2019; Yoon et al., 2013). The AdeABC efflux pump was confirmed to export not only tigecycline but also beta-lactam, fluoroquinolone, aminoglycoside and chloramphenicol (Coyne et al., 2011; Xu et al., 2019). As mentioned in previous studies, the overexpression of AdeABC efflux pump has been shown to be related to amino acid substitutions in AdeRS TCS (Gerson et al., 2018; Yoon et al., 2013). Our results indicated that the two amino acid substitutions in AdeRS TCS may be associated to the overexpression of AdeABC efflux pump. S16N is in the transmembrane domain of AdeS, while T137S is in the receiver domain of AdeR. Further structural and biochemical studies are needed to unravel how the two amino acid substitutions affect AdeRS TCS signal transmission.

Although, the CRAn isolates were collected from six major medical centers in Taiwan, one of the limitations in our study is that such small sample sizes may not represent the genotype distribution of CRAn in the world. Another limitation is that the contribution of potential mechanism other than efflux pump was not examined. On the basis of our current data, we suggest that Type 1 AdeRS TCS could directly stimulate the expression of the AdeABC efflux pump and is associated with eravacycline and omadacycline resistance. Thus, Type 1 AdeRS TCS could be a potential target to screen inhibitors as tetracycline derivative adjuvants for future combination therapy.

## Conclusion

In conclusion, eravacycline and omadacycline MICs of CRAn were correlated closely with those of tigecycline and eravacycline. They demonstrated improved potency over tigecycline against populations of CRAn, including Tn-CRAn isolates. The synergistic activity of NMP on eravacycline and tigecycline was observed, but not on omadacycline. The type 1 AdeRS TCS pattern was able to reduce the susceptibilities of the three antibiotics against *A. nosocomialis* by the regulation of AdeABC efflux pumps medicated by AdeRS TCS.

## Members of the ACTION Study Group

The members of the ACTION study group include Yea-Yuan Chang (National Yang-Ming University Hospital, Yilan, Taiwan), Y-SY (Division of Infectious Diseases and Tropical Medicine, Department of Internal Medicine, Tri-Service General Hospital, National Defense Medical Centre, Taipei, Taiwan), Chung-Ting Chen (Department of Emergency Medicine, Taipei Veterans General Hospital, Taipei, Taiwan), Yuag-Meng Liu (Changhua Christian Hospital, Changhua, Taiwan), Shu-Chen Kuo (National Institute of Infectious Diseases and Vaccinology, National Health Research Institute, Maoli County, Taiwan), Chang-Pan Liu (MacKay Memorial Hospital, Taipei, Taiwan), Te-Li Chen (Graduate Institute of Life Sciences, National Defense Medical Centre, Taipei, Taiwan), and Y-TL (Taipei Veterans General Hospital, Taipei, Taiwan).

## Data Availability Statement

The raw data supporting the conclusions of this article will be made available by the authors, without undue reservation.

## Author Contributions

Y-TL, Y-SY, Y-CC, T-YC, W-JH, and I-CL conceptualized the study, performed the experiments, and analyzed the data. J-RS, H-YC and Y-TL was responsible for funding acquisition, aided in conceptualization of the study, and writing of the manuscript. All authors contributed to the article and approved the submitted version.

## Conflict of Interest

The authors declare that the research was conducted in the absence of any commercial or financial relationships that could be construed as a potential conflict of interest.

## Footnotes

1 https://online.phyloviz.net/index
