# Supplementary material for: AdeABC Efflux Pump Controlled by AdeRS Two Component System Conferring Resistance to Tigecycline, Omadacycline and Eravacycline in Clinical Carbapenem Resistant Acinetobacter nosocomialis
Source: Front Microbiol. 2020 Nov 2;11:584789. doi: 10.3389/fmicb.2020.584789 (PMC7667285; doi:10.3389/fmicb.2020.584789)

**Table S1.** Antimicrobial susceptibilities of Tn-CRAn isolates.

| **Antimicrobial agents** | **Breakpoint (mg/L)** | |  | **MIC (mg/L)** | | |  | **Interpretation of susceptibility, n (%)** | | |
| --- | --- | --- | --- | --- | --- | --- | --- | --- | --- | --- |
|  | **susceptible** | **resistant** |  | **Range** | **MIC_50_** | **MIC_90_** |  | **susceptible** | **intermediate** | **resistant** |
| **Amikacin** | ≦ 16 | ≧ 64 |  | 16 – (≧64) | ≧64 | ≧64 |  | 11 (19.3) | 1 (1.8) | 45 (78.9) |
| **Gentamicin** | ≦ 4 | ≧ 16 |  | 8- (≧16) | ≧16 | ≧16 |  | 0 | 2 (3.5) | 55 (96.5) |
| **Cefepime** | ≦ 8 | ≧ 32 |  | 16- (≧32) | (≧32) | (≧32) |  | 0 | 3 (5.3) | 54 (94.7) |
| **Ceftazidime** | ≦ 4 | ≧ 32 |  | 8 - (≧32) | (≧32) | (≧32) |  | 0 | 13 (22.8) | 44 (77.2) |
| **Ciprofloxacin** | ≦ 1 | ≧ 4 |  | 1 - (≧4) | ≧4 | ≧4 |  | 2 (3.5) | 0 | 55 (96.5) |
| **Colistin** | ≦ 2 | ≧ 4 |  | 0.25 –(≧4) | 0.25 | 0.25 |  | 53 (93.0) | 0 | 4 (7.0) |
| **Imipenem** | ≦ 2 | ≧ 8 |  | 8- (≧16) | ≧16 | ≧16 |  | 0 | 0 | 57 (100) |
| **Tigecycline** | ≦ 2 | ≧ 8 |  | 4-32 | 8 | 8 |  | 0 | 26 (45.6) | 31 (54.4) |
| **Piperacillin-tazobactam** | ≦ 16/4 | ≧ 128/4 |  | ≧ 128/4 | ≧ 128/4 | ≧ 128/4 |  | 0 | 0 | 57 (100) |
| **Trimethoprim/sulfamethoxazole** | ≦ 2/38 | ≧ 4/76 |  | ≧ 4/76 | ≧ 4/76 | ≧ 4/76 |  | 0 | 0 | 57 (100) |

**Figure S1**. The genetic relationship of Tn-CRAn isolates based on MLST data. Numbers inside each circle indicate the sequence type (ST) types. The size of each circle corresponds to the number of isolates. The line connecting the circles indicates the relationship between different STs.


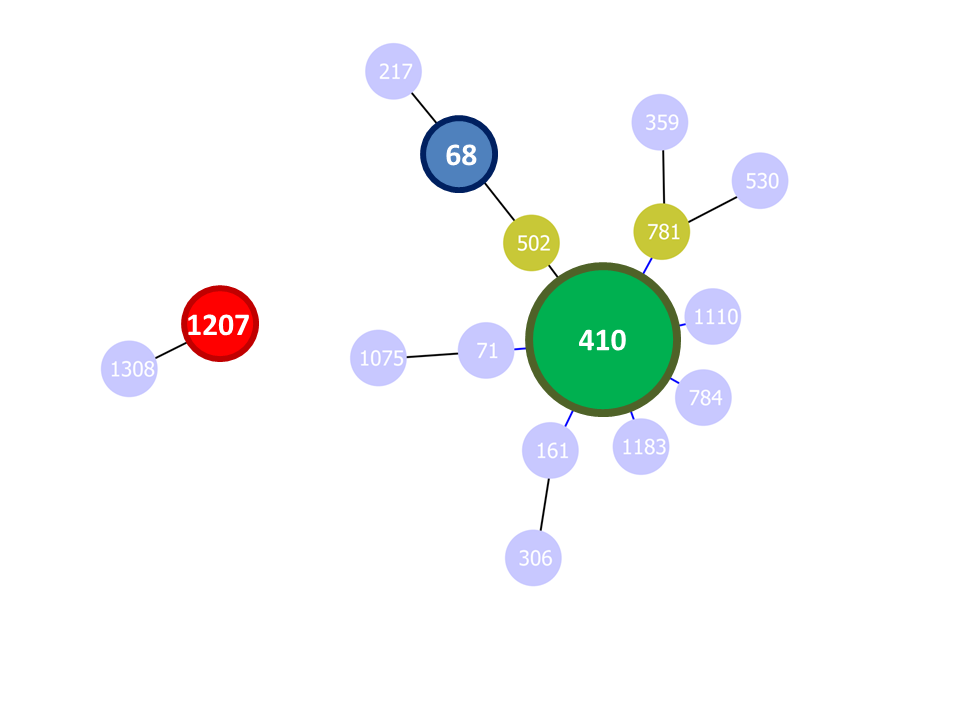


Figure S2. Protein sequence alignment of AdeR and AdeS. Stars indicate identical amino acids. Dots indicate different amino acids.

1. AdeR


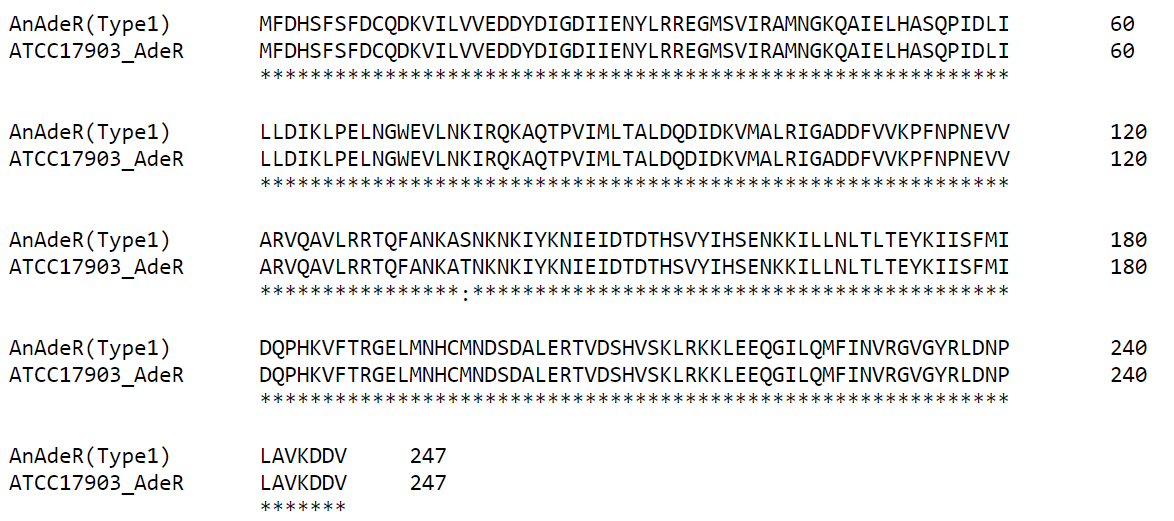


1. AdeS


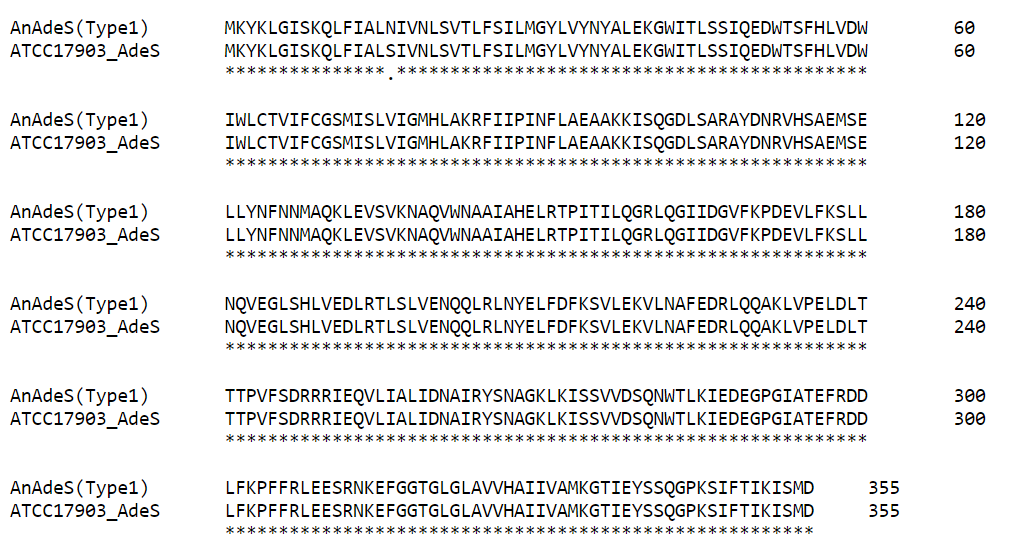

Supplement: Supplementary file 1 [file Data_Sheet_1.docx]
